# Acute Severe Aortic Regurgitation Post-PCI

**DOI:** 10.1016/j.jaccas.2024.103101

**Published:** 2025-02-05

**Authors:** Robert Ambrogetti, Gareth Squire, Nick Harvey, Amerjeet Banning, Andrew Ladwiniec, Gavin J. Murphy, Iain Squire

**Affiliations:** a Department of Cardiology, Glenfield Hospital, University Hospitals of Leicester NHS Trust, Leicester, United Kingdom; b Department of Anaesthesia, Glenfield Hospital, University Hospitals of Leicester NHS Trust, Leicester, United Kingdom; c Department of Cardiovascular Sciences and the National Institute for Health and Care Research, Leicester Biomedical Research Centre, Glenfield Hospital, University of Leicester and University Hospitals of Leicester NHS Trust, Leicester, United Kingdom

**Keywords:** aortic regurgitation, aortic valve replacement, coronary angiography, echocardiography, iatrogenic, primary coronary intervention, rotational atherectomy

## Abstract

The ability to manage more complex coronary disease has evolved with the development of new technologies, techniques, and practitioner experience. The increasing technical difficulty of interventional procedures is known to be associated with an increased risk of complications. This can include coronary perforation, coronary dissection, coronary thrombosis, and aortic valve dysfunction. Permanent aortic valve damage caused by guide catheter, wire instrumentation or stent migration is a rare occurrence. We report the case of a woman in her 50s who developed acute severe aortic regurgitation requiring aortic valve replacement post–percutaneous coronary intervention.

## Graphical Abstract

**Figure:**
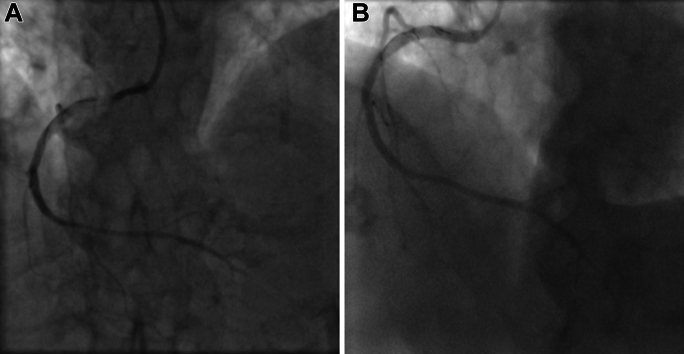

## History of Presentation

A woman in her 50s was admitted to cardiology after presenting with unstable angina. Diagnostic coronary angiography revealed a severe calcific lesion in the mid left anterior descending (LAD) coronary artery, a severe lesion in the proximal to mid right coronary artery (RCA), and a severe lesion in a small caliber obtuse marginal posterior left branch of the left circumflex coronary (LCx) artery. The left main stem was unobstructed. A decision was made to proceed to percutaneous coronary intervention (PCI) on the LAD and RCA with medical management of the LCx, because it was considered too small for intervention or grafting.

**Take-Home Messages**

• Physicians should maintain a high index of suspicion for complications postprocedure, especially when faced with a deteriorating patient.
• This case highlights the critical importance of physical examination and appropriate use of imaging in acutely unwell patients postprocedure.

Via a right radial approach through a 6-F sheath, the LCA was engaged with a 6-F 3.5-VL catheter, and the LAD wired with a Sion Blue. The procedure was intravascular ultrasound–guided and involved intravascular lithotripsy for calcium modification, with successful deployment of 2 drug-eluting stents. Next, the RCA was engaged with a 6-F JR4 guide catheter. The vessel was wired with a Sion Blue. However, a balloon could not be passed across the lesion. The Sion Blue wire was exchanged for a rota floppy wire via a caravel microcatheter to facilitate rotational atherectomy. A 1.5-mm RotaPro burr was unable to pass. In the process of changing to a smaller burr, the guide catheter and wire position were lost. At this point, it was noted that the rota wire had fractured and remained in the RCA at the location of the lesion. The RCA was re-engaged with a 6-F AL 0.75 guide catheter, and the lesion was rewired with a Sion Blue and exchanged for a second rota floppy wire via a Turnpike spiral microcatheter. Rotablation was successfully performed with a 1.25-mm rota burr followed by a 1.5-mm rota burr. The rota floppy wire was then exchanged for a Sion Blue wire, and the lesion was further prepared with a 2.5 × 15 NC Emerge balloon. Following this, a 2.5 × 38-mm Xience Pro-S drug-eluting stent was placed distally, and a 3.0 × 28-mm Xience pro-S stent was placed proximally, with high pressure 1:1 postdilatation. The intention was that the stented segment effectively jailed the residual fractured rota wire within the RCA. intravascular ultrasound confirmed good stent expansion with the fractured wire jailed behind the stent. A bedside echocardiogram immediately after the procedure showed no evidence of pericardial effusion or valvular dysfunction.

**Figure 1:**
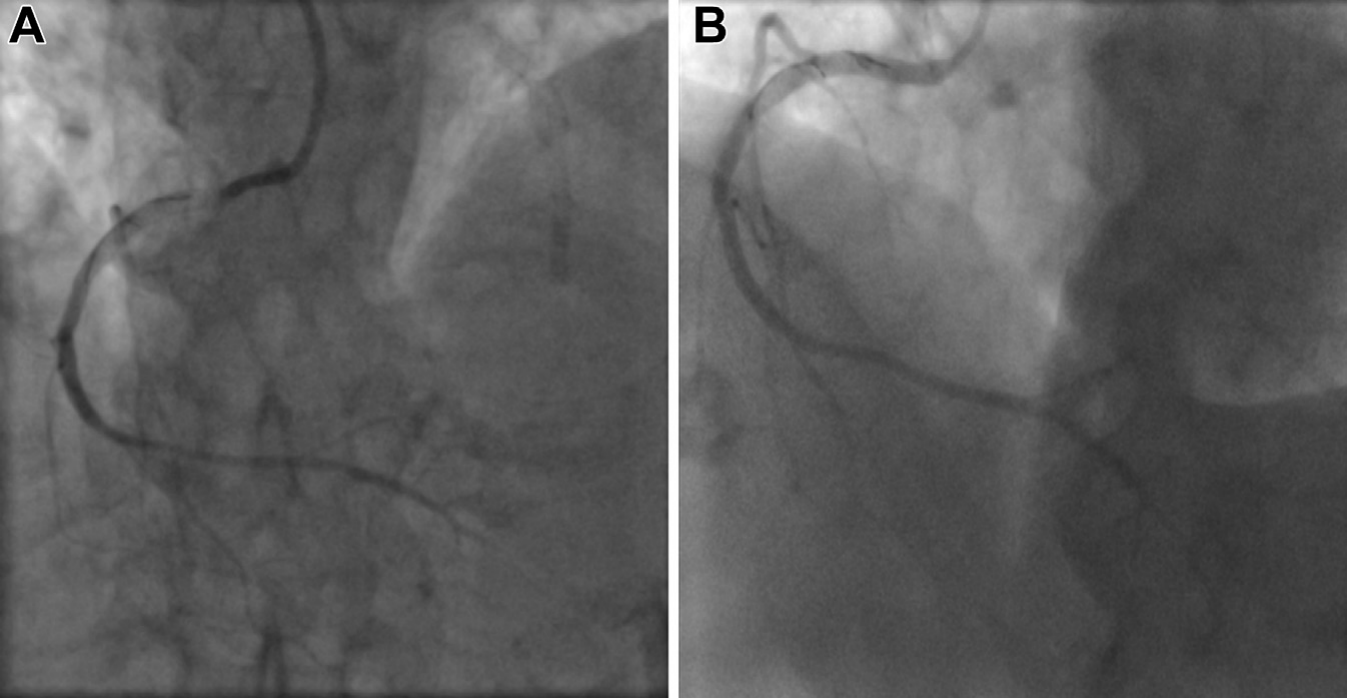
Coronary Angiography Fluoroscopic Images (A) Rota wire position in right coronary artery post–first angiography. (B) Rota wire in right coronary artery post–second angiography.

Later that day, the patient was taken back to angiography because of a reoccurrence of chest pain and ST-segment depression in leads II, III, and AVF on electrocardiogram. At repeat angiography, the LAD and RCA stents were patent and free of obvious complications. Angiographically, the fractured wire did not appear to have migrated (Figure 1). The lesions in the obtuse marginal and posterior left ventricular branch of the LCx branches were unchanged from the previous PCI. Considering these findings, the patient was transferred back to the coronary care unit.

Over the next 48 to 72 hours, the patient developed an increasing oxygen requirement, dyspnea, and tachypnea. On examination, her jugular venous pressure was elevated to the angle of the jaw, the radial pulse was classically collapsing with a change in pulse pressure from 55 mm Hg preoperatively to 79 mm Hg (blood pressure 135/56 mm Hg), and a low-pitched early diastolic murmur was heard in the aortic area.

## Past Medical History

The patient had a past medical history of type 2 diabetes, hypertension, and asthma.

## Differential Diagnosis

The differential diagnosis of dyspnea and increasing oxygen requirement post-PCI is broad. With evidence of a new diastolic murmur, acute heart failure secondary to aortic regurgitation (AR) was the primary differential, with possible etiologies including procedural-related aortic dissection, valvular damage, and a new ischemic event. Other causes such as arrhythmia, pulmonary embolism, asthma exacerbation, and chest infection were considered less likely given the clinical examination findings.

## Investigations

**Figure 2:**
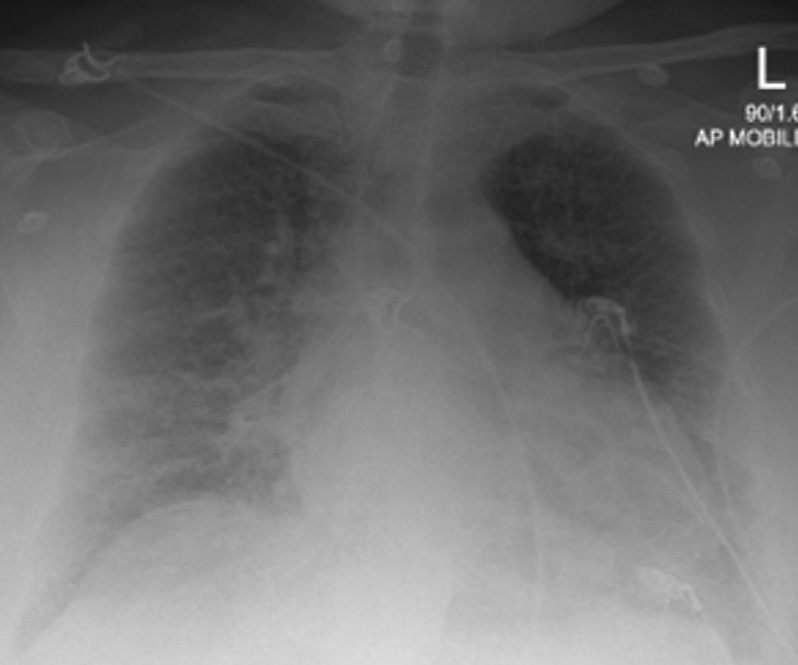
Chest X-Ray Showing Cardiomegaly and Pulmonary Edema

Her chest x-ray showed cardiomegaly, pulmonary edema, and increased interstitial markings bilaterally (Figure 2).

Bedside echocardiogram demonstrated AR, which was at least moderate in severity. A computed tomography (CT) aorta was requested in the context of a differential diagnosis of catheter-induced aortic dissection. This was subsequently excluded on CT aorta. The following day clinical examination, performed by the same senior clinician, suggested worsening AR; repeat transthoracic echocardiogram showed a broad, turbulent jet of AR now graded as severe. A linear, hyperechoic structure with a metallic appearance was identified protruding through the aortic valve into the left ventricular outflow tract (apical 5- and 3-chamber views) (Videos 1 and 2).

**Figure 3:**
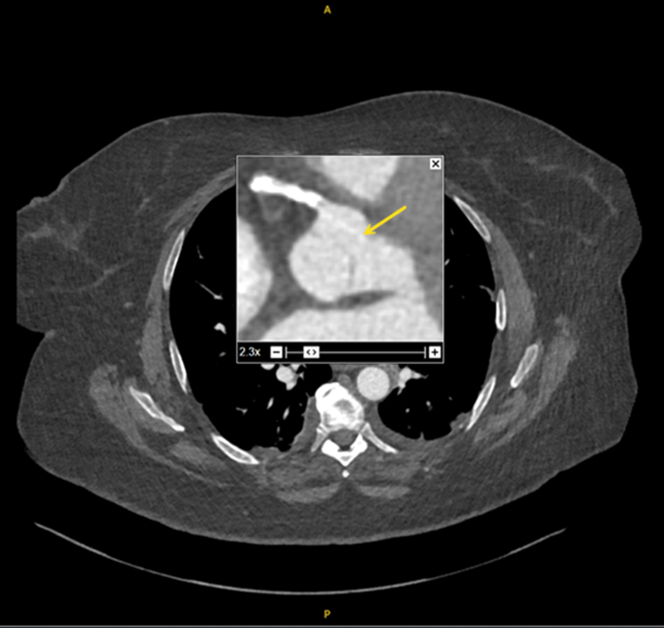
Computed Tomography Aorta Showing Subtle Hyperattenuating Linear Artefact Arising in the Right Coronary Artery Progressing Across the Right Coronary Cusp Suggesting a Protruding Wire

A second retrospective review of the CT-aorta demonstrated a subtle hyperattenuating linear artefact arising in the RCA and running across the RCC (Figure 3).

## Management

The patient was started on intravenous diuretic agents. Despite optimal medical management, the patient continued to decline over the next 24 hours. Hence, the patient was referred for cardiac surgery opinion and underwent emergency aortic valve replacement the same day. The patient had a successful 21-mm ON–X mechanical aortic valve replacement via open heart surgery with an intraoperative transesophageal echocardiogram (TOE).

**Figure 4:**
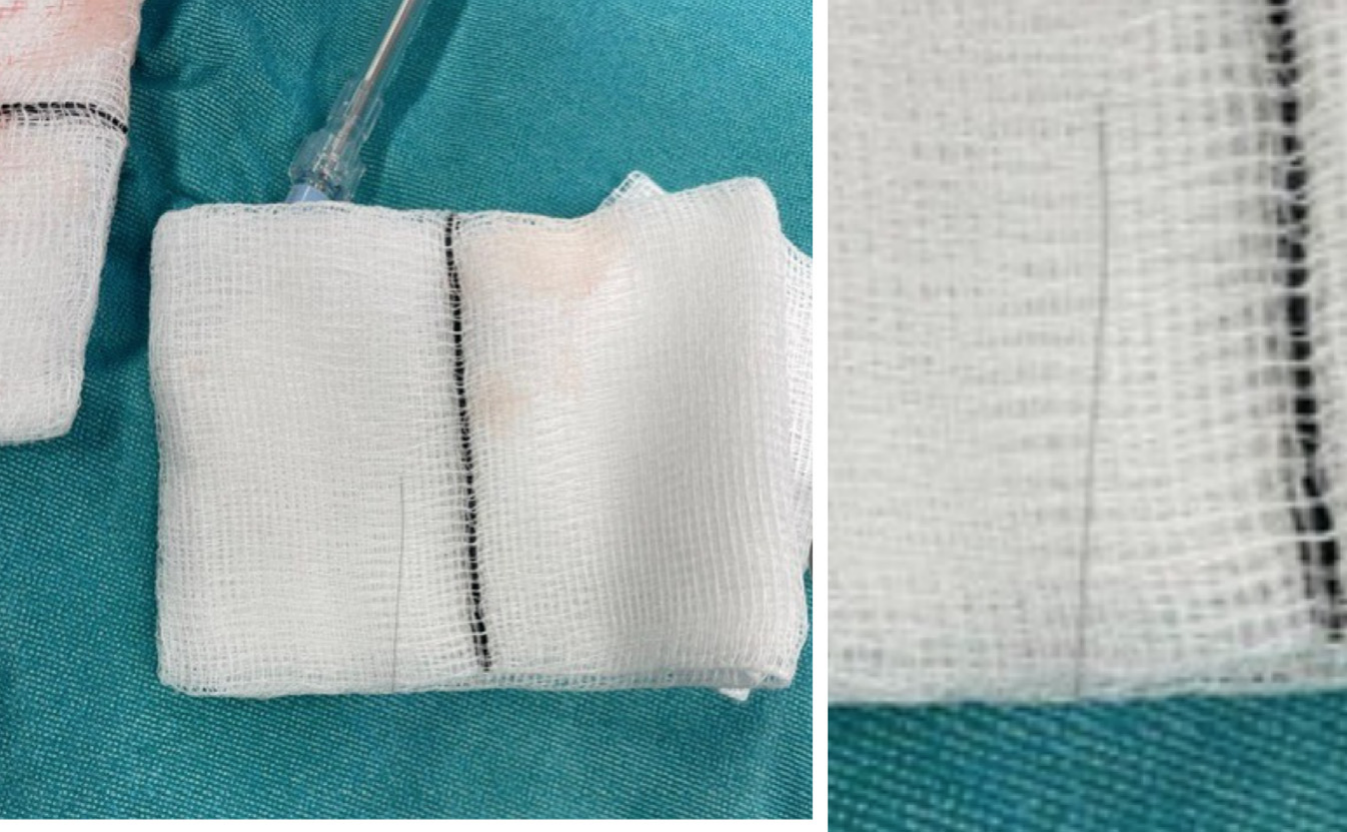
Rota Wire Piercing the Aortic Valve Was Removed at Surgery

No perioperative or postoperative complications were noted. Intraoperatively, the wire (Figure 4) was protruding from the RCA and had pierced through the right coronary cusp and into the LVOT. This was also observed on intraoperative TOE images (Video 3, Video 4, Video 5, Video 6).

## Outcome and Follow-Up

The patient had an uneventful stay in intensive care postoperatively, being extubated on day 2 and stepped down to the ward on day 3. After a period of observation, medication titration, and inpatient rehabilitation, the patient was discharged home on triple therapy and outpatient follow-up.

## Discussion

Despite its widespread implementation and high success rate, PCI has associated risks of minor and major complications.^1^ Acute severe AR is an exceedingly rare post-PCI complication with a reported incidence of roughly 1:10,000.^2^

Most previously reported cases of acute severe AR attributed to direct aortic valve damage at PCI present acutely.^3^ Most of these cases, which present acutely, have been associated with damage caused by a catheter or wire at PCI.^3^ Those cases attributed to stent protrusion or migration have been associated with a delayed onset of at least 1 month or more from PCI.^3^, ^4^, ^5^ Previous cases associated with rotablation resulted in the identification of significant AR ultimately requiring surgical aortic valve replacement.^6^ Similarly, our case was identified 3 days post-PCI. Our patient reported symptoms after the initial procedure, suggesting that the AR progressively led to heart failure over several days. In our case, the presence of a new characteristic diastolic murmur narrowed the differential diagnosis and directed investigations. This highlights the importance of thorough physical examination in the diagnostic process of post-PCI complications.

Guide catheter disengagement and resultant rota burr contact with the aortic cusps has been previously suggested as a potential mechanism of aortic valve damage in complex procedures.^6^ At the transition between the radio-opaque and non–radio-opaque sections of the wire, there is a transition from 0.228 to 0.355 mm at the distal segment of the wire. This transition point is susceptible to being sheared off by the rota burr, leaving a residual fractured distal end. However, this may not explain an extended segment of wire protruding into the LVOT, as seen in this case. A rota wire forming a loop may create a more proximal point of wire susceptible to injury from the rota burr, accounting for a more extended segment of retained wire. Direct damage from the catheter or wires are other possible mechanisms of injury leading to AR.

Rota wires have a diameter (0.35 mm) below that of conventional CT aorta protocol slice thickness (∼1 mm).^7^ As a result, rota wires can be missed or easily overlooked as artefacts on CT, angiography, or intravascular ultrasound when ruling out aortic pathology as the cause of AR, as in our case. In most cases, the diagnosis was either confirmed on TOE or at surgery. In our case, a new murmur and transthoracic echocardiographic evidence of AR were noted days after PCI. The position of the wire into the LVOT was later confirmed at surgery. Hence, iatrogenic aortic valve injury should be kept in the differential of a patient deteriorating within the first 10 days post-PCI, especially in the presence of a new diastolic murmur and echocardiographic evidence of AR.

Most cases have been refractory to medical treatment and eventually require surgical intervention in the form of either aortic valve replacement or repair.^3^^,^^6^ Importantly, surgical intervention is generally associated with good outcomes, with most patients being discharged after uneventful postoperative periods, as seen in our case.^3^^,^^6^

## Conclusions

AR caused by instrumentation at PCI is rare. Developments in equipment, technology, and practitioner experience have made more complex PCI possible. In turn, physicians should maintain a high index of suspicion for novel iatrogenic complications, especially when faced with a deteriorating patient postprocedure. General careful management of guide catheters and intraoperative equipment can reduce the risk of iatrogenic complications, especially in complex procedures. This case highlights the importance of physical examination and echocardiography in prompt diagnosis. All reported cases of AR post-PCI, including our case, required surgical intervention, mainly in the form of aortic valve replacement. Prompt identification and surgical intervention have been associated with a good prognosis in most cases.

## Funding Support and Author Disclosures

The authors have reported that they have no relationships relevant to the contents of this paper to disclose.

## Appendix

Video 1 Transthoracic echocardiogram 3- and 5-chamber views showing hyperechoic linear structure protruding through the aortic valve into the left ventricular outflow tract.

Video 2 Transthoracic echocardiogram 3- and 5-chamber views showing hyperechoic linear structure protruding through the aortic valve into the left ventricular outflow tract.

Video 3 Transesophageal echocardiogram showing wire protruding through the aortic valve into the LVOT. TOE SAX view, lacerated right coronary cusp with flail segment.

Video 4 TOE SAX view, color Doppler demonstrating free aortic regurgitation.

Video 5 TOE PLAX view, lacerated right coronary cusp with flail segment

Video 6 TOE PLAX view, color Doppler, eccentric posteriorly directed aortic regurgitation through a flail right coronary cusp.
